# Supplementary material for: The effect of dumping syndrome severity on health-related quality of life among bariatric surgery patients in Saudi Arabia
Source: PLoS One. 2026 Mar 23;21(3):e0345638. doi: 10.1371/journal.pone.0345638 (PMC13008100; doi:10.1371/journal.pone.0345638)
Supplement: S1 Table — (DOCX) [file pone.0345638.s001.docx]

S1 Table. Descriptive analysis of the patients perceived interference and troubles associated with DS symptoms, n = 237

|  | **Total sample  (n = 237)** | **Early DS  (n = 182)** | **Late DS  (n = 55)** | **Comparison between subgroups** |
| --- | --- | --- | --- | --- |
| **Perceived Interference from DS symptoms (1-4 scale)** | | | | |
| How troublesome is Desire to lie down | 2.08 ± 0.90 | 2.13 ± 0.93 | 1.91 ± 0.79 | p = 0.159^T^ |
| How troublesome is Weakness | 2.61 ± 0.96 | 2.58 ± 0.98 | 2.70 ± 0.88 | p = 0.503^T^ |
| How troublesome is Sleepiness | 2.25 ± 1.00 | 2.28 ± 1.02 | 2.15 ± 0.93 | p = 0.494^T^ |
| How troublesome is Palpitation | 2.72 ± 1.02 | 2.72 ± 1.03 | 2.71 ± 1.01 | p = 0.963^T^ |
| How troublesome is Restlessness | 2.85 ± 1.01 | 2.87 ± 1.01 | 2.81 ± 1.03 | p = 0.796^T^ |
| How troublesome is Dizziness | 2.75 ± 1.00 | 2.75 ± 1.00 | 2.75 ± 0.99 | p = 0.991^T^ |
| How troublesome is Feeling warm, Sweat, Clammy | 2.61 ± 1.09 | 2.62 ± 1.15 | 2.61 ± 0.92 | p = 0.970^W^ |
| How troublesome is Nausea | 2.85 ± 1.03 | 2.88 ± 1.00 | 2.72 ± 1.10 | p = 0.379^T^ |
| How troublesome is Vomiting | 2.83 ± 1.08 | 2.89 ± 1.07 | 2.68 ± 1.12 | p = 0.287^T^ |
| How troublesome is Abdominal cramps | 2.64 ± 1.01 | 2.68 ± 1.04 | 2.49 ± 0.93 | p = 0.302^T^ |
| Overall Perceived troubles and interference with Dumping syndrome score | 2.43 ± 0.84 | 2.45 ± 0.85 | 2.40 ± 0.80 | p = 0.714^T^ |
| Severity of symptoms  None-Very Low^1^  Moderate^2^  Severe^3^ | 8 (3.4%)  87 (36.7%)  142 (59.9%) | 6 (3.3%)  67 (36.8%)  109 (59.9%) | 2 (3.6%)  20 (36.4%)  33 (60.0%) | p = 1.00^F^ |
| Patient intervened these symptoms  No  Yes | 77 (32.5%)  160 (67.5%) | 65 (35.7%)  117 (64.3%) | 12 (21.8%)  43 (78.2%) | p = 0.054^C^ |
| Intervention undertaken  Visited surgeon who performed operation  Visited another physician  Went to the pharmacist  Turned to non-medical methods (Internet, family and friends, etc.) | (n = 160)  100 (62.5%)  59 (36.9%)  41 (25.6%)  96 (60.0%) | (n = 117)  70 (59.8%)  34 (29.1%)  29 (24.8%)  69 (59.0%) | (n = 43)  30 (69.8%)  25 (58.1%)  12 (27.9%)  27 (62.8%) | p = 0.250^C^  **p < 0.001^C^**  p = 0.689^C^  p = 0.662^C^ |
| Patient received health education regarding DS  No  Yes | 113 (47.7%)  124 (52.3%) | 86 (47.3%)  96 (52.7%) | 27 (49.1%)  28 (50.9%) | p = 0.811^C^ |

Note: reported values are frequency (%) or Mean ± SD

^C^ chi-square test, ^F^ Fisher’s Exact test, ^T^ independent samples t-test, ^W^ Welch t-test (for unequal variances)

^1^ None-very low: No symptom that’s troublesome

^2^ Moderate: at least one symptom that is little to a bit troublesome

^3^ Severe: at least two symptoms with quite a bit to very much troublesome
