# Supplementary material for: The effect of dumping syndrome severity on health-related quality of life among bariatric surgery patients in Saudi Arabia
Source: PLoS One. 2026 Mar 23;21(3):e0345638. doi: 10.1371/journal.pone.0345638 (PMC13008100; doi:10.1371/journal.pone.0345638)
Supplement: Attachment — Submitted filename: Response to Reviewers.docx [file pone.0345638.s002.docx]

**Response letter:**

**Journal Requirements:**

**1. When submitting your revision, we need you to address these additional requirements.**

Please ensure that your manuscript meets PLOS ONE's style requirements, including those for file naming. The PLOS ONE style templates can be found at

https://journals.plos.org/plosone/s/file?id=wjVg/PLOSOne_formatting_sample_main_body.pdf and

https://journals.plos.org/plosone/s/file?id=ba62/PLOSOne_formatting_sample_title_authors_affiliations.pdf

**Response:**

Thank you for your comment. The file names were updated as requested. The manuscript was edited and formated by a proffesional editor and the journal’s templet was used (please see attched editing certificate).

**2**. **Thank you for stating the following in the Acknowledgments Section of** **your manuscript:**

Special thanks for support by the College of Medicine Research Center, Deanship of Scientific Research, King Saud University Riyadh, Saudi Arabia. We also acknowledge the use of the generative AI tool ChatGPT to support language review and proofreading in the preparation of some sections of this manuscript.

We note that you have provided funding information that is not currently declared in your Funding Statement. However, funding information should not appear in the Acknowledgments section or other areas of your manuscript. We will only publish funding information present in the Funding Statement section of the online submission form.

Please remove any funding-related text from the manuscript and let us know how you would like to update your Funding Statement. Currently, your Funding Statement reads as follows:

The author(s) received no specific funding for this work.

Please include your amended statements within your cover letter; we will change the online submission form on your behalf.

**Response:**

Thank you for your comment. This study did not receive any funding. Therefore, there is no need to change the funding statement in the manuscript.

**3**. **Please provide a complete Data Availability Statement in the submission form, ensuring you include all necessary access information or a reason for why you are unable to make your data freely accessible. If your research concerns only data provided within your submission, please write " All data are in the manuscript and/or supporting information files " as your Data Availability Statement.**

**Response:**

Thenak youu for your comment. The satemnt “All data are in the manuscript and/or supporting information files” was added as requested on page no. 19, lines 396-397.

**4**. **Please upload a new copy of Figure 1 as the detail is not clear. Please follow the link for more information: https://journals.plos.org/plosone/s/figures**

**Response:**

Thank you for your comment. Figure 1 and 2 were enhanced and uploaded.

**5. Please include captions for your Supporting Information files at the end of your manuscript**, and update any in-text citations to match accordingly. Please see our Supporting Information guidelines for more information: http://journals.plos.org/plosone/s/supporting-information.

**Response:**

Thank you for your comment. The supporting information section was added in the manuscript on page no.25, lines 525-532 and the in-text citations were updated accordingly.

**6**. **If the reviewer comments include a recommendation to cite specific previously published works, please review and evaluate these publications to determine whether they are relevant and should be cited. There is no requirement to cite these works unless the editor has indicated otherwise.**

**Response:**

Thank you for your comment. References were updated after considering the reiewer’s comment.

**7**. **Please review your reference list to ensure that it is complete and correct**. If you have cited papers that have been retracted, please include the rationale for doing so in the manuscript text, or remove these references and replace them with relevant current references. Any changes to the reference list should be mentioned in the rebuttal letter that accompanies your revised manuscript. If you need to cite a retracted article, indicate the article’s retracted status in the References list and also include a citation and full reference for the retraction notice.

**Response:**

Thank you for your comment. The reference list was updated.

[Note: HTML markup is below. Please do not edit.]

**Comments to the Author**

1. Is the manuscript technically sound, and do the data support the conclusions?

The manuscript must describe a technically sound piece of scientific research with data that supports the conclusions. Experiments must have been conducted rigorously, with appropriate controls, replication, and sample sizes. The conclusions must be drawn appropriately based on the data presented.

Reviewer #1: Yes

Reviewer #2: Partly

2. Has the statistical analysis been performed appropriately and rigorously?

Reviewer #1: Yes

Reviewer #2: I Don't Know

3. Have the authors made all data underlying the findings in their manuscript fully available?

The [PLOS Data policy](http://www.plosone.org/static/policies.action#sharing) requires authors to make all data underlying the findings described in their manuscript fully available without restriction, with rare exception (please refer to the Data Availability Statement in the manuscript PDF file). The data should be provided as part of the manuscript or its supporting information, or deposited to a public repository. For example, in addition to summary statistics, the data points behind means, medians and variance measures should be available. If there are restrictions on publicly sharing data—e.g. participant privacy or use of data from a third party—those must be specified.

Reviewer #1: Yes

Reviewer #2: Yes

4. Is the manuscript presented in an intelligible fashion and written in standard English?

PLOS ONE does not copyedit accepted manuscripts, so the language in submitted articles must be clear, correct, and unambiguous. Any typographical or grammatical errors should be corrected at revision, so please note any specific errors here.

Reviewer #1: Yes

Reviewer #2: Yes

5. Review Comments to the Author

Please use the space provided to explain your answers to the questions above. You may also include additional comments for the author, including concerns about dual publication, research ethics, or publication ethics. (Please upload your review as an attachment if it exceeds 20,000 characters)

**Reviewer #1:** The study evaluated the impact of early and late dumping syndrome (DS) on health-related quality of life (HRQoL) in patients undergoing bariatric surgery in Saudi Arabia, including both sleeve gastrectomy (SG) and Roux-en-Y gastric bypass (RYGB). Considering the epidemic proportions of obesity and the need for its treatment, it brings necessary and important information in the postoperative management of patients. The results revealed a significant impairment of physical and mental health, with low mean scores of the physical (PHS) and mental (MHS) components. The severity of symptoms was a major determinant of the decrease in HRQoL, with patients with severe DS presenting significantly lower scores compared to those with moderate forms. In both early DS and late DS, over 60% of participants reported severe symptoms, the most common being the need to lie down and nausea. The differences in clinical manifestation between the two types of DS influenced the behavior of seeking medical care, with patients with late DS requesting non-surgical consultations more frequently, probably due to persistent neuroglycopenic symptoms. The study provides valuable regional data and emphasizes the need for early identification and effective management of DS to improve postoperative quality of life.

**Response:**

Thank you for your encourging comments.

**Reviewer #2:** Dear Authors,
I read your manuscript carefully. I have some questions and comments and I hope addressing them, can increase the quality of your upcoming manuscript.

**1- There is a mismatch between methods (February 2024 until June 2024) and results (between February 2024 and December 2024) in data collection times. Please correct.**

**Response:**

We thank the reviewer for their invaluable input. The method section was reviweed and the duration was corrected to be from February 2024 to December 2024 (page no.4, line 87).

**2- You mentioned that "Participants without DS (n = 115) were excluded entirely from the analysis." It may prone your study to the selection bias and limit comparing HRQoL between DS and non-DS patients, and make DS as an independent factor for HRQoL questionnaire.**

**Response:**

We appreciate the reviewer’s thoughtful comment regarding potential selection bias. Our primary objective was to investigate the determinants and gradients of HRQoL within the DS population (e.g., how the severity and timing of early vs. late DS to specific QoL domains), rather than to compare DS and non-DS patients or to establish DS as an independent determinant of HRQoL at the population level.

We agree that excluding participants without DS (n = 115) restricts formal comparison of HRQoL between DS and non-DS groups and may limit external generalizability. This is now acknowledged as a limitation in the manuscript (page no. 18, lines 372-374). Nonetheless, the observed HRQoL impairments in our symptomatic DS cohort are aligned with prior studies showing that patients with moderate-to-severe dumping symptoms have substantially lower HRQoL scores than asymptomatic or minimally symptomatic post-gastrectomy controls, supporting the clinical relevance of our within-DS findings.

**3- The Results indicate no significant difference between early and late DS in several analyses, so please avoid overstating subgroup differences.**

**Response:**

Thank you for your comment. The results were reviewed and the overstating sentence comparing early and late DS was deleted (page no.11 , lines 225-226).

**4- I suggest using a similar study to assess the severity of DS after RYGB in the discussion part(PMID: 36607445).**

**Response:**

We thank the reviewer for highlighting this study (PMID: 36607445). The main objective of that paper was to evaluate the effect of dumping syndrome (DS) severity on weight loss outcomes after Roux-en-Y gastric bypass (RYGB) in patients with class III obesity. In contrast, our study focuses on assessing health-related quality of life among adult patients in Saudi Arabia with varying DS severity after either sleeve gastrectomy or RYGB.

Given these fundamental differences in primary outcomes (weight loss vs quality of life), patient population, and inclusion of two different bariatric procedures in our work, we believe the study is not directly comparable as a methodological model for our research. However, we agree it remains relevant to cite in the discussion as supportive evidence that DS severity can have clinically meaningful consequences after bariatric surgery, while emphasizing that our study extends this literature by addressing patient-reported quality of life across different surgical techniques in a Middle Eastern population (page no. 17, lines 350-352).

6. PLOS authors have the option to publish the peer review history of their article ([what does this mean?](https://journals.plos.org/plosone/s/editorial-and-peer-review-process#loc-peer-review-history)). If published, this will include your full peer review and any attached files.


If you choose “no”, your identity will remain anonymous but your review may still be made public.


**Do you want your identity to be public for this peer review?** For information about this choice, including consent withdrawal, please see our [Privacy Policy](https://www.plos.org/privacy-policy).

Reviewer #1: No

Reviewer #2: No

[NOTE: If reviewer comments were submitted as an attachment file, they will be attached to this email and accessible via the submission site. Please log into your account, locate the manuscript record, and check for the action link "View Attachments". If this link does not appear, there are no attachment files.]

To ensure your figures meet our technical requirements, please review our figure guidelines: https://journals.plos.org/plosone/s/figures

You may also use PLOS’s free figure tool, NAAS, to help you prepare publication quality figures: https://journals.plos.org/plosone/s/figures#loc-tools-for-figure-preparation.

NAAS will assess whether your figures meet our technical requirements by comparing each figure against our figure specifications.
